# A Method of High Throughput Monitoring Crop Physiology Using Chlorophyll Fluorescence and Multispectral Imaging

**DOI:** 10.3389/fpls.2018.00407

**Published:** 2018-03-28

**Authors:** Heng Wang, Xiangjie Qian, Lan Zhang, Sailong Xu, Haifeng Li, Xiaojian Xia, Liankui Dai, Liang Xu, Jingquan Yu, Xu Liu

**Affiliations:** ^1^State Key Laboratory of Modern Optical Instrumentation, Zhejiang University, Hangzhou, China; ^2^Department of Horticulture, Zhejiang University, Hangzhou, China; ^3^College of Control Science and Engineering, Zhejiang University, Hangzhou, China

**Keywords:** chlorophyll fluorescence imaging, multispectral imaging, high throughput, crop physiology, drought, nutrition deficiency, plant disease, monitoring system

## Abstract

We present a high throughput crop physiology condition monitoring system and corresponding monitoring method. The monitoring system can perform large-area chlorophyll fluorescence imaging and multispectral imaging. The monitoring method can determine the crop current condition continuously and non-destructively. We choose chlorophyll fluorescence parameters and relative reflectance of multispectral as the indicators of crop physiological status. Using tomato as experiment subject, the typical crop physiological stress, such as drought, nutrition deficiency and plant disease can be distinguished by the monitoring method. Furthermore, we have studied the correlation between the physiological indicators and the degree of stress. Besides realizing the continuous monitoring of crop physiology, the monitoring system and method provide the possibility of machine automatic diagnosis of the plant physiology.

**Highlights:** A newly designed high throughput crop physiology monitoring system and the corresponding monitoring method are described in this study. Different types of stress can induce distinct fluorescence and spectral characteristics, which can be used to evaluate the physiological status of plants.

## Introduction

Improvement of agricultural production capacity is extremely important to human beings in face of the expanding population in the 21st century (Alston et al., 2009; Godfray et al., 2010). To achieve this goal, we need modernization of agriculture which relies on breakthrough in aspects such as dynamic monitoring, intelligent control, and automatic implementation (Klukas et al., 2014; Sankaran et al., 2015). For nursery of high quality seedlings, monitoring the crop growing status continuously and non-destructively is the first step to make decisions as to change the environment conditions, and adjust the inputs of water and nutrients (White et al., 2012; Andrade-Sanchez et al., 2014). It is also necessary to obtain the growth status information to guide the control of plant diseases in greenhouse and field (Stafford, 2000; Diacono et al., 2013). However, it is challenging to monitor the physiology of a population of crops in a large scale and continuously over a period of time. Automatic approach is required for detection of large quantities of plants. Meanwhile, the information about the crop growth status should be visualized and quantified in order to offer a more intuitive and standardized reference for management of the environment (Berger et al., 2010; Virlet et al., 2014).

To detect the physiological status of plants, a range of technologies and/or their combinations have been applied (Chaerle et al., 2007a), such as chlorophyll fluorescence imaging (Barbagallo et al., 2003; Baker and Rosenqvist, 2004), multispectral imaging (Lenk et al., 2007; Zarco-Tejada et al., 2009), thermal imaging (Kaukoranta et al., 2005; Chaerle et al., 2007b) and terahertz technique (Born et al., 2014). However, the extension of these approaches for large-scale use is not straightforward. The data volume acquired is not large enough when the techniques mentioned above are applied in greenhouse, field and other places of large area. Importantly, the small amount of data cannot fully reflect the overall physiological status of crop population.

Among different methods for detecting plant physiology, chlorophyll fluorescence is used as an accurate probe of photosynthesis. In the process of photosynthesis, the light energy absorbed by chlorophyll converts to photochemical energy stored as ATP and NADPH through a series of reactions (Baker, 2008). Most of the light energy is used for photosynthesis, and parts of the light energy are dissipated as heat or reemitted as fluorescence. Changes in photosynthesis and heat dissipation resulted in fluctuation of the chlorophyll fluorescence. Measurement of chlorophyll fluorescence can be divided into non-modulated and modulated modes (Govindje, 1995; Stirbet and Govindjee, 2011). With the development of light emitting diodes (LED) and saturating pulse technique, the modulated chlorophyll fluorescence measurement has been more widely used. For commercial use, instruments used in different situation have been produced by manufacturers such as Walz^1^, Photon Systems Instruments^2^, Hanstech Instruments^3^ and so on. There are also some research institutes have developed setups of chlorophyll fluorescence measurement (Oxborough and Baker, 1997; Lichtenthaler and Babani, 2000; Nedbal et al., 2000; Porcarcastell et al., 2008; Kalaji et al., 2012). The typical technical parameters such as even illumination, saturating pulse intensity and detection area are the main concerns of researchers (Murchie and Lawson, 2013).

Multispectral imaging of plant is applied from microscopic scale to remote sensing (Dammer et al., 2011; Laliberte et al., 2011; Huang et al., 2015). The reflectance of different wavelengths is associated with the chemical composition of leaves, e.g., the content of chlorophyll and nutrient elements (Yendrek et al., 2017). Analysis based on reflectance curve or reflectance image has been applied in related research (Chaerle et al., 2007c; Bauriegel et al., 2011). Imaging has the inherent advantage of showing the distribution and variation of signals over a sample. To achieve imaging under different wavelengths, CCD cameras combined with different filters are usually used.

In this study, we developed a system for high throughput monitoring the crop physiology states in a greenhouse. The system employs the monitoring method integrating chlorophyll fluorescence imaging with multispectral imaging. Using a greenhouse crop tomato as the research object, we set up typical environmental stresses including drought, nutrition deficiency and plant disease to study the application of our system in the monitoring of greenhouse crop physiology. The method combining chlorophyll fluorescence imaging with multispectral imaging can help us comprehensively judge different crop stresses. Through selecting appropriate parameters of chlorophyll fluorescence and relative reflectance of multispectral as the indicators of crop physiological status, the type of stress suffered by crops can be distinguished. In addition, we attempted to set up calibration of the indicators of crop physiological status, which can be used as reference to estimate the general stress degree of plants. Furthermore, automatic diagnosis of the plant physiology by the system can be implemented based on the method.

## Materials and Methods

### Plant Material

Seedlings of tomato (*Solanum lycopersicum* L. cv. Hezuo 903) were used. Seeds were germinated in a growth medium filled with a mixture of peat and vermiculite (3:1, v/v) in trays in a growth chamber. Seedlings (except those for nutrition experiments) at two-leaf stage were transplanted into plastic pots (15 cm diameter and 15 cm depth, one seedling per pot) containing peat and vermiculite (3:1, v/v). The two-leaf stage seedlings for nutrition experiments were transplanted into plastic pots containing vermiculite and perlite (3:1, v/v). All seedlings were watered and fertilized with Hoagland’s solution (Hoagland, 1950) every 2 days. The growth conditions were as follows: 12-h photoperiod, temperature of 25°C/20°C (day/night), and photosynthetic photon flux density of 600 μmol m^-2^ s^-1^.

### Drought Treatment

When tomato seedlings were at four-leaf stage, drought experiment was performed. The day when we started withdrawing water was set as day 1. Soil relative water content was determined by using a soil moisture meter (Takeme, GREYWELL, CHN).

### Nutrition Deficiency Treatment

When tomato seedlings were at four-leaf stage, nitrogen deficiency experiment was performed. The treatment group was watered with nitrogen deficient nutrient solution. The Hoagland’s nutrient solution contained 354 mg/L Ca(NO_3_)_2_⋅4H_2_O, 505.5 mg/L KNO_3_, 115 mg/L NH_4_H_2_PO_4_ and 246.5 mg/L MgSO_4_⋅7H_2_O, and nitrogen deficient nutrient solution contained 166.5 mg/L CaCl_2_, 348.5 mg/L K_2_SO_4_, 136 mg/L KH_2_PO_4_, and 246.5 mg/L MgSO_4_⋅7H_2_O. The day when the nutrient solution was replaced was set as day 1.

### Disease Treatment

When tomato seedlings were at five- to six-leaf stage, the plants were inoculated with *Botrytis cinerea* (BO5-10 strain) suspensions at a density of 2 × 10^5^ spores per mL (Zhang et al., 2015). The day when the seedlings were inoculated with *B. cinerea* was set as day 1.

### Different Nitrogen Strength Treatment

When tomato seedlings were at four-leaf stage, they were separated into three groups. Plants of the three groups were, respectively, watered with nitrogen deficiency, full strength and double strength (708 mg/L Ca(NO_3_)_2_⋅4H_2_O, 1011 mg/L KNO_3_, 230 mg/L NH_4_H_2_PO_4_, and 246.5 mg/L MgSO_4_⋅7H_2_O) Hoagland’s solution. The day beginning different nitrogen strength treatment was set as day 1. From day 1 to day 7, tomato leaves were sampled for chlorophyll fluorescence measurement and total nitrogen determination.

### Monitoring System

**FIGURE 1:**
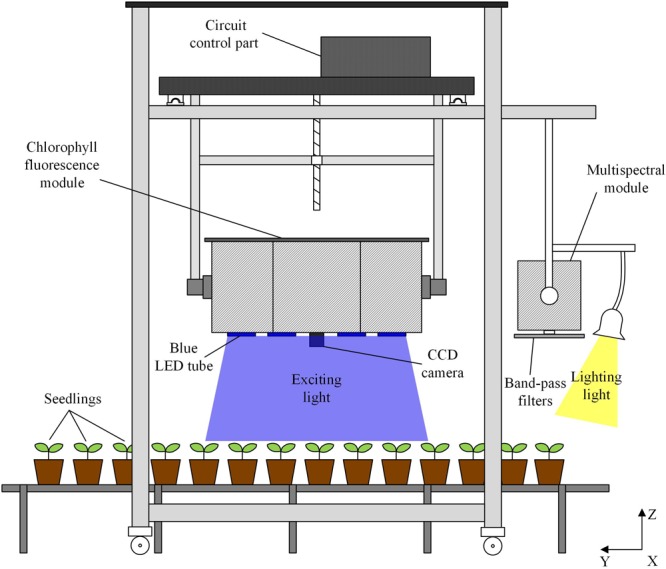
Schematic diagram of crop physiology monitoring system (see **Supplementary Figure S1** for the system photo in working state). Chlorophyll fluorescence module provides uniform illumination within the field of view of the fluorescence camera. Imaging acquisition, including the control of the camera and the light source by the circuit part as well as data analysis is carried out by the software developed on the computer. Through scanning along with x, y, z axes, the monitoring of all the plants on the seedling bed can be accomplished by the system.

The monitoring of plant physiology is carried out by the system integrating chlorophyll fluorescence imaging module and multispectral imaging module (**Figure 1**). The chlorophyll fluorescence imaging module consists of lighting part, CCD (PCO 1300, PCO, GER) imaging part and circuit control part. The lighting part contains 16 × 100 W LEDs to provide actinic light for activating the photosynthesis, saturating light pulse for inhibiting photosynthesis and excitation light pulse for measuring the fluorescence signal. The central wavelength of the LED is 460 nm. Square light pipe is used to even LED lighting spot. LED lighting spots focus on the same region to form 0.45 m × 0.34 m uniform illumination area. The CCD camera with a cut-off high-pass filter to detect fluorescence above 650 nm is surrounded by the 16 lighting tubes. The fluorescence images are captured by the signal pulse generated by the control circuit. For multispectral imaging, CCD (PCO 1300, PCO, GER) is placed behind band-pass filters. The band-pass filters are set on a rotating plate. With the rotating of the plate, CCD captures images under different wavelengths. Incandescent lamps are used to provide visible wavelengths lighting condition.

### Monitoring Method and Data Analysis

A software supporting modulated chlorophyll fluorescence measurement is developed based on Visual Studio 2010 (Microsoft, United States). In order to avoid the interference of ambient light, plants were dark adapted for at least half an hour at night before chlorophyll fluorescence measurement. First, the minimal level of fluorescence *F*_o_ was obtained after the measuring light (0.1 μmol photons m^-2^ s^-1^) was opened. Next, the maximal level of fluorescence *F*_m_ was obtained when the photosynthesis was temporarily inhibited by a saturating light pulse (>6000 μmol photons m^-2^ s^-1^). Then, the actinic light (200 μmol photons m^-2^ s^-1^) was opened to simulate the ambient light condition, under which the photosynthesis of the plant leaves was activated. When the photosynthesis was stable, the steady-state fluorescence *F* was obtained, meanwhile a saturating light pulse was applied to elicit the maximal level of fluorescence *F*_m_′ under light condition.

After chlorophyll fluorescence measurement of all plants on the locations of the seedling bed, the monitoring system went back to the initial position and acquired multispectral images of the same locations. Through two-dimensional scanning on the field, the monitoring system can achieve measurement in a larger scale.

In order to distinguish the fluorescence and spectral characteristic induced by drought, nitrogen deficiency, and plant disease (gray leaf mold), we choose three parameters to reflect the physiological status of plants. *F*_v_/*F*_m_, the maximum quantum efficiency of photosystem II (PSII), represents the potential maximum capacity of PSII reaction center transforming the photon energy absorbed by PSII to photochemical energy. Φ_PSII_ represents the operating quantum efficiency of PSII. 550/510, the ratio of 550 nm spectral reflectance to 510 nm spectral reflectance, represents the degree of greening of the leaves.

Image processing and data analysis is carried out using Matlab 2017b (Mathworks, United States). Resolution of images acquired is 696 × 520 and plant area should be segmented from the images. For chlorophyll fluorescence parameter images, the parameter of the non-plant area is very small as the non-fluorescing of non-plant area. So, we set threshold to segment the plant area. For multispectral images, we implemented an algorithm based on K-means clustering (Macqueen, 1965; Lucchese and Mitra, 2001), in which image can be grouped into two classes representing the plant and the background, respectively. Based on the four chlorophyll fluorescence images *F*_o_, *F*_m_, *F, F*_m_′, we can calculate the maximum quantum efficiency of PSII *F*_v_/*F*_m_ = (*F*_m_–*F*_o_)/*F*_m_ and operating quantum efficiency of PSII Φ_PSII_ = (*F*_m_′–*F*)/*F*_m_′. Based on the spectral images of 550 nm and 510 nm, we can calculate the relative reflectance 550/510.

### Determination of Water Potential

Leaf water potential (Begg and Turner, 1970) of drought-stressed plants was measured with a water potential meter (WP4C, Decagon Devices, United States). Canopy leaves of plants under different drought condition were cut and placed inside the sample cup. The water potential was determined by the dew point method.

### Determination of Total Nitrogen

The total nitrogen is determined spectrophotometrically using the Nessle’s reagent. The first procedure is the digestion of the sample. Fresh plant leaves were dried to constant weight and ground to a fine power with mortar and pestle. Sample powder (0.1 g) was placed in a boiling tube. H_2_SO_4_ (5 mL) was added to the boiling tube and then the tube was placed overnight. All boiling tubes with the samples were heated in graphite digestion apparatus and the temperature was raised from 150 to 200°C until the appearance of white smoke. Ten drops of 30% H_2_O_2_ was added when the solution became dark brown and mixed the solution constantly. Then the solution was heated for 5 min. The H_2_O_2_ treatment and heating was repeated 3–5 times till the remained H_2_O_2_ was removed and the solution was clear and transparent. Then the solution was diluted to 50 mL with double distilled water (ddH_2_O).

After pretreatment procedure, 1 mL sample solution was mixed with 2 mL 25% sodium potassium tartrate, 2 mL Nessler’s reagent (HgI_2_, KI, and NaOH) and 45 mL non-ammonia water. The absorbance of the resulting solution at 480 nm (OD_480_) was measured by spectrophotometer (TU-1900, PERSEE, CHN). For standard solution, 10 mg/L (NH_4_)_2_SO_4_ was prepared. Calibration curve was obtained with OD_480_ of 0, 2, 4, 6, 8, and 10 mL standard solution. By comparison with the calibration curve, the total nitrogen of the sample was determined.

## Results

### The Fluorescence and Spectral Characteristics of Tomato Plants Under Drought Stress

**FIGURE 2:**
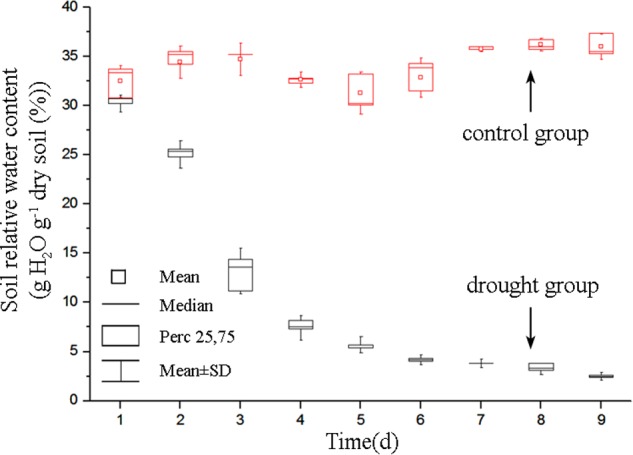
Dynamics of soil relative water content of the drought group and the control group. For each line, *n* = 5.

**FIGURE 3:**
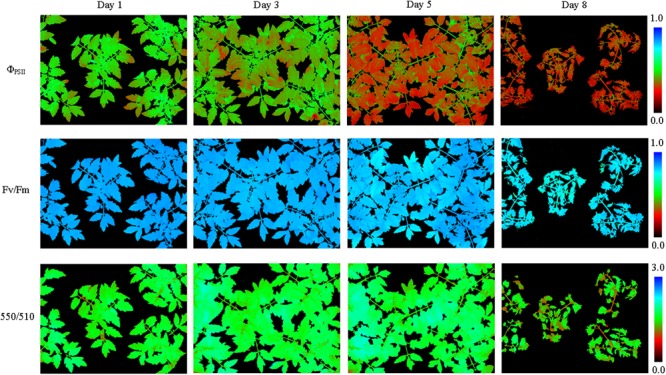
Pseudo-color images of Φ_PSII_, *F*_v_/*F*_m_,and 550/510 parameters of tomatoes under drought stress (see **Supplementary Figure S2** for more images). From day 1 to day 5 plant morphology didn’t change notably, and Φ_PSII_ of all the plant leaves decreased notably. As the drought degree deepened, plant leaves wilted and *F*_v_/*F*_m_ and 550/510 decreased obviously.

**FIGURE 4:**
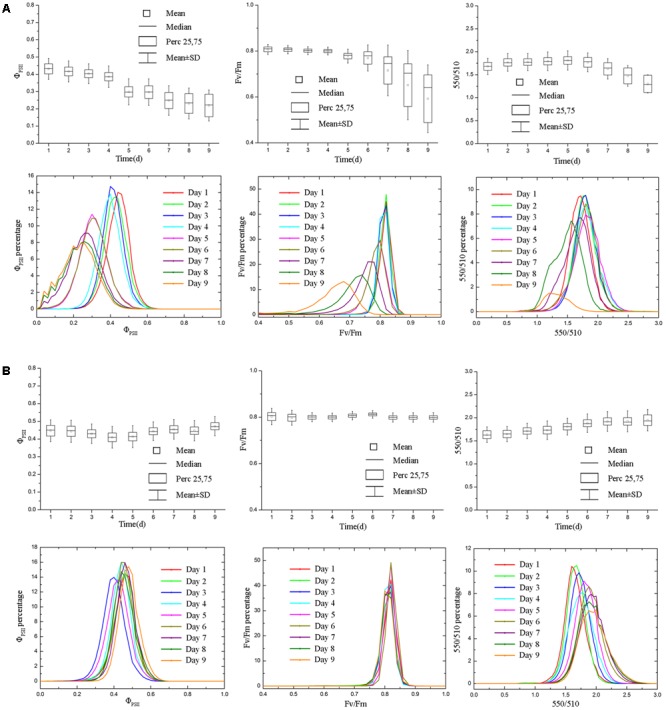
Boxplot and distribution of Φ_PSII_, *F*_v_/*F*_m_ and 550/510 parameters of tomatoes under drought stress. Panel **(A)** and **(B)** represent data of drought stress and control group, respectively. Φ_PSII_ responded more rapidly to drought stress **(A)**, whereas *F*_v_/*F*_m_ and 550/510 parameters showed declines in the later stage of experiment (day 7 – day 9), when the soil relative water content was quite low. For tomato plants in control group **(B)**, Φ_PSII_, *F*_v_/*F*_m_, and 550/510 remained stable (Φ_PSII_ and *F*_v_/*F*_m_) or showed the rising trend (550/510).

The soil relative water content decreased dramatically from day 1 to day 5 after withdraw water (**Figure 2**). In the later stage of experiment, the soil relative water content was relatively stable but at a low level. Pseudo-color images of Φ_PSII_, *F*_v_/*F*_m_ and 550/510 parameters (**Figure 3**) visualize how drought stress affected the physiological status of tomato plants (see **Supplementary Figures S2, S3** for more images). As soil relative water content decreased, mean Φ_PSII_ declined steadily from above 0.4 on day 1 to about 0.3 on day 5 (**Figure 4A**). When plants suffered from severe drought stress (day 7 – day 9), tomato leaves wilted and the Φ_PSII_ did not decrease further, remaining about 0.25. Until this time, both *F*_v_/*F*_m_ and 550/510 showed significant declines. Therefore, Φ_PSII_ is a sensitive indicator to show changes in plant physiology during the initial stage of drought stress when the plant morphology didn’t change obviously. For plants in the well-watered control group, Φ_PSII_ and *F*_v_/*F*_m_ remained stable around 0.4 and 0.8, respectively. Meanwhile, the value of 550/510 showed a slight rising trend (**Figure 4B**).

### The Fluorescence and Spectral Characteristics of Tomato Plants Under Nutrition Deficiency Stress

**FIGURE 5:**
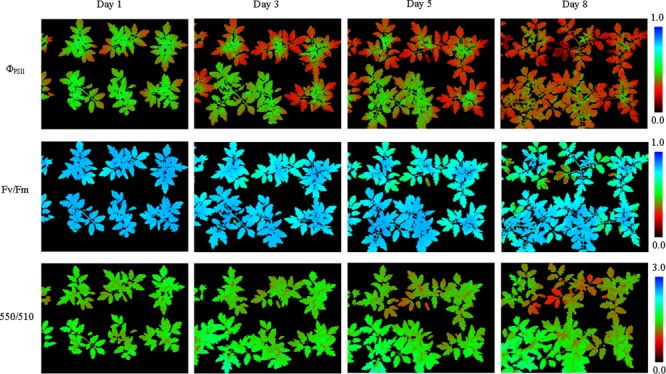
Pseudo-color images of Φ_PSII_, *F*_v_/*F*_m_ and 550/510 parameters of tomato plants under nitrogen deficiency stress (see **Supplementary Figure S4** for more images). Nitrogen deficiency at first caused declines of Φ_PSII_, *F*_v_/*F*_m_, and 550/510 parameters in the old leaves. As the degree of stress increased, declines of parameters showed in the new leaves.

**FIGURE 6:**
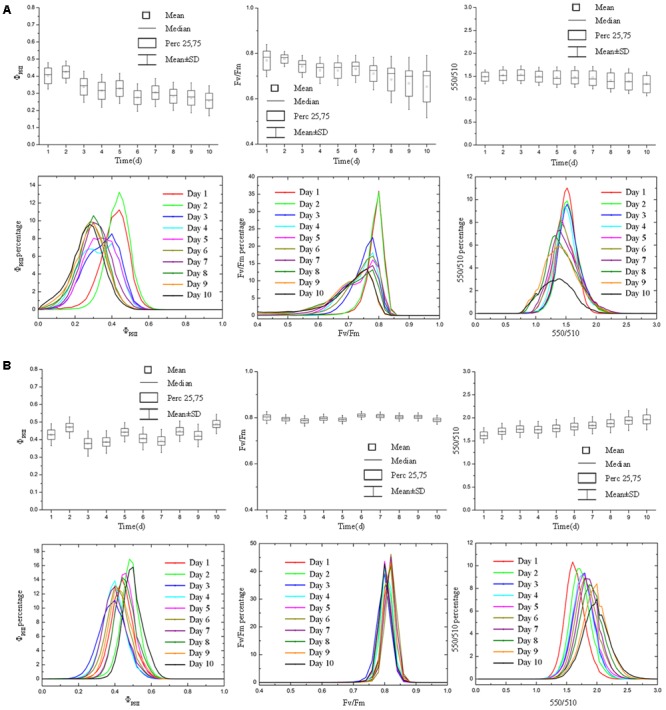
Boxplot and distribution of Φ_PSII_, *F*_v_/*F*_m_, and 550/510 parameters of tomato plants under nitrogen deficiency stress. Panel **(A)** and **(B)** represent data of nitrogen deficiency stress and control group, respectively. For tomato plants under nitrogen deficiency stress **(A)**, Φ_PSII_, *F*_v_/*F*_m_, and 550/510 declined as the degree of nitrogen deficiency increased. The Φ_PSII_, *F*_v_/*F*_m_, and 550/510 of old and new leaves responded differentially to the nitrogen deficiency stress, leading to an increase in the parameter standard deviation, especially for *F*_v_/*F*_m_. For tomato plants in control group **(B)**, Φ_PSII_, *F*_v_/*F*_m_ and 550/510 remained stable (Φ_PSII_ and *F*_v_/*F*_m_) or showed the rising trend (550/510).

From pseudo-color images of Φ_PSII_, *F*_v_/*F*_m_, and 550/510 parameters (**Figure 5**), we can see how nitrogen deficiency affected physiology of tomato plants (see **Supplementary Figures S4, S5** for more images). As the degree of nitrogen deficiency increased, Φ_PSII_, *F*_v_/*F*_m_, and 550/510 all showed declining trend (**Figure 6A**). The decreases of parameters first appeared in old leaves and then spread to new leaves. This resulted in an increase in parameter standard deviation, especially for *F*_v_/*F*_m_. For tomato plants in the control group, Φ_PSII_ and *F*_v_/*F*_m_ remained around 0.4 and 0.8, respectively, and 550/510 showed a rising trend above 1.5 (**Figure 6B**).

### The Fluorescence and Spectral Characteristics of Tomato Plants Under Disease Stress

**FIGURE 7:**
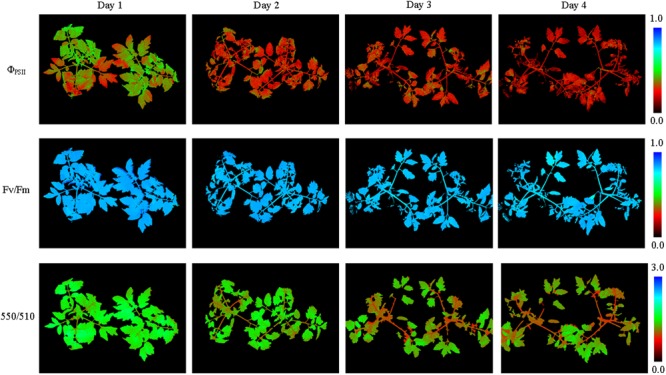
Pseudo-color images of Φ_PSII_, *F*_v_/*F*_m_, and 550/510 parameters of tomato plants infected with *Botrytis cinerea*. As the infection deepened, plant leaves curled downward and Φ_PSII_ decreased most obviously.

**FIGURE 8:**
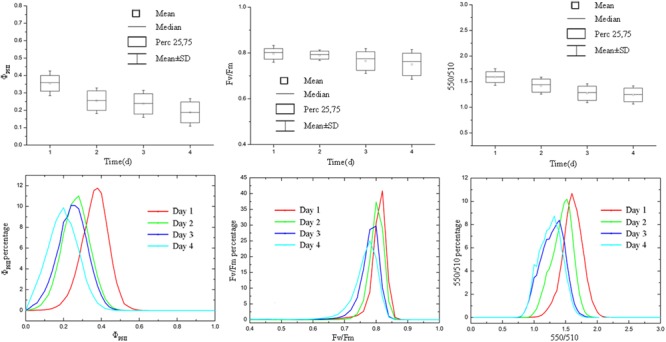
Boxplot and distribution of Φ_PSII_, *F*_v_/*F*_m_, and 550/510 parameters of tomato plants infected with *B. cinerea*. Φ_PSII_, *F*_v_/*F*_m_, and 550/510 declined as the disease symptom aggravated.

From pseudo-color images of Φ_PSII_, *F*_v_/*F*_m_, and 550/510 parameters (**Figure 7**), we can see how *B. cinerea* infection affected tomato plants (see **Supplementary Figure S6** for more images). As the disease symptom aggravated, Φ_PSII_, *F*_v_/*F*_m_, and 550/510 all showed significant decline (**Figure 8**). The decline rate is faster than that of nitrogen deficiency. The plant morphology changed a lot, as shown by the curled downward infected leaves.

### Correlation Between Φ_PSII_ and Water Potential

**FIGURE 9:**
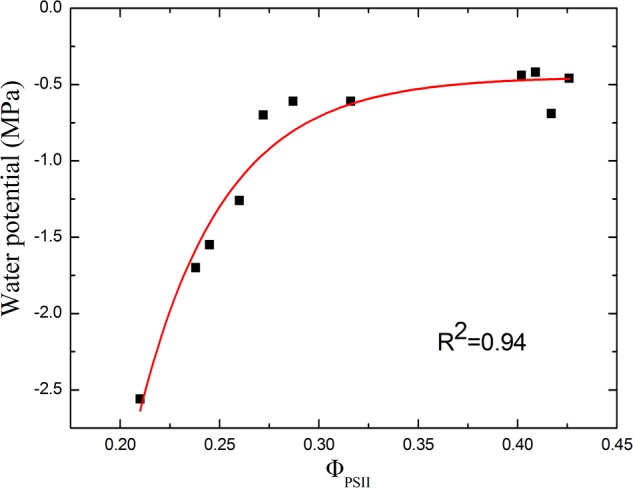
Correlation between Φ_PSII_ and water potential of tomato leaves in response to drought stress. As the correlation curve shows, Φ_PSII_ correlates well with the water potential in the early stage of drought stress. However, under severer drought condition, Φ_PSII_ changes little as the water potential declines.

For drought stress, both soil relative water content and Φ_PSII_ declined steadily during the first few days. Therefore, by sampling tomato leaves in different drought degree, we studied the correlation between water potential and Φ_PSII_ of tomato leaves (**Figure 9**). We can see the non-linear relationship between Φ_PSII_ and the water potential. When the drought is not severe, Φ_PSII_ is sensitive to the changes in water potential. Φ_PSII_ varies from 0.43 to 0.28, when water potential is in the range of -0.5∼-1.0 MPa. Plants experiencing a threshold levels of water deficiency (water potential <-1.0 MPa) displays considerable decrease in Φ_PSII_. However, for leaves which have water potential -1.0 to -2.5 MPa, Φ_PSII_ only declines from 0.28 to 0.21. This correlation between Φ_PSII_ and water potential suggests that rapid decline of Φ_PSII_ is a sensitive indicator of initial drought stress. As a non-invasive means of identifying the degree of drought stress, Φ_PSII_ can be potentially used to monitor the plant growth status, especially for water saving irrigation.

### Correlation Between *F*_v_/*F*_m_ and Total Nitrogen Content

**FIGURE 10:**
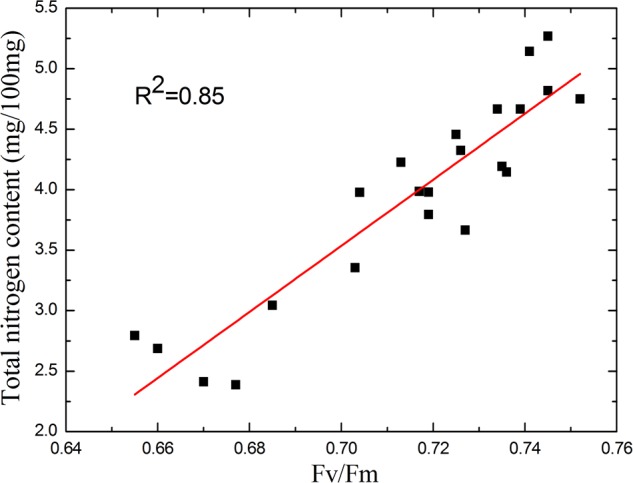
Correlation between *F*_v_/*F*_m_ and total nitrogen content. As the correlation curve shows, *F*_v_/*F*_m_ linearly reflects the nitrogen content. The sensitivity of *F*_v_/*F*_m_ to the nitrogen deficiency remains as the total nitrogen content substantially decreased.

When the plants suffer nutrient deficiency, both Φ_PSII_ and *F*_v_/*F*_m_ decline with the progression of the stress (Lu and Zhang, 2000; Damatta et al., 2002). In contrast to the early stage of drought stress, the decline of *F*_v_/*F*_m_ is more significant in response to nutrient deficiency. The increase in standard deviation of *F*_v_/*F*_m_ (**Figure 6A**) also shows *F*_v_/*F*_m_ is representative. So, by sampling tomato leaves with different nitrogen strength treatment, we studied the correlation between *F*_v_/*F*_m_ and total nitrogen content of tomato leaves. As shown in **Figure 10**, the correlation between *F*_v_/*F*_m_ and total nitrogen content is high with a coefficient of determination of 0.85. In other words, *F*_v_/*F*_m_ can linearly reflect the nitrogen content.

## Discussion

In this paper, we introduce a newly developed crop physiology detection system and monitoring method. The main goal of our study is to provide a sensitive and non-destructive tool to continuously monitor crop physiology status in a large scale, providing the basis for selection of stress-tolerant germplasm and for related management work.

### Indicators of Crop Physiology

Plant physiology diagnosis based on imaging has advantage of high visibility and large data volume. We try to analyze the images with simple point of view, and further to prepare for the machine automatic diagnosis. We choose chlorophyll fluorescence and multispectral parameters as the indicators to monitor the crop physiology status.

Drought, nitrogen deficiency and disease are the three types of crop stress frequently occur in agriculture. At least three indicators are required to characterize the stresses. Chlorophyll fluorescence parameters Φ_PSII_ and *F*_v_/*F*_m_ are sensitive to the plant physiology and are widely used. We choose them as two indicators, and we choose the third indicator from multispectral images. In traditional measurement of multispectral parameters, absolute reflectivity is often used and the calibration board is necessary. However, the use of calibration board is not convenient for the system structure and large area measurement. Thus, relative reflectance between different wavelengths has been used in this study. The ratio change of different spectral composition can reflect the change of leaf chroma caused by the composition of the leaves. Meanwhile, the use of relative reflectance decreases the requirement of the light source for spatial domain uniformity.

By comparing with the control groups, we can identify the threshold of stressful conditions. Φ_PSII_ is sensitive to plant physiological conditions, and has fluctuation even in the control group. Under lighting condition of 200 μmol m^-2^ s^-1^ photosynthetic photon flux density, the normal range of Φ_PSII_ should be around 0.4. When Φ_PSII_ decreases close to 0.3, the plants may be in some stressed conditions. The fluctuation of *F*_v_/*F*_m_ is relative small and the normal range of *F*_v_/*F*_m_ should be around 0.8. When *F*_v_/*F*_m_ decreases below 0.75, PSII reaction center has been affected. For 550/510, it increases with the accumulation of chlorophyll. The normal range of 550/510 should be above 1.5.

### Distinct Fluorescence and Spectral Characteristics Induced by Different Stresses

We found that different types of stresses can induce distinct changes of chlorophyll fluorescence and multispectral signals, which can be used as indicators of plant physiology. For the drought stress, stomatal closure results in the decline of photosynthetic rate and the decrease in Φ_PSII_. Φ_PSII_ is proportional to the carbon assimilation rate. In general, Φ_PSII_ decreases in response to different stresses (Baker, 2008). Drought stress does not affect *F*_v_/*F*_m_ sharply when the plants are not in extreme drought condition (Giardi et al., 1996; Woo et al., 2008). A long-term drought stress will cause a decrease in *F*_v_/*F*_m_, potentially due to a secondary nutrient stress. Therefore, Φ_PSII_ can show the early warning of drought, and we use Φ_PSII_ to evaluate the degree of drought stress.

*F*_v_/*F*_m_ was commonly used for assessing plant performance under stress (Bresson et al., 2015; Awlia et al., 2016). Nitrogen deficiency affects PSII photochemistry and decreases the quantum yield of PSII electron transport. The decline of *F*_v_/*F*_m_ is mainly due to the decrease of the maximal fluorescence *F*_m_ (Lu and Zhang, 2000). The photoinhibition caused by nitrogen deficiency is associated with the inactivation of PSII reaction centers. In addition, nitrogen deficiency induced a significant decrease in CO_2_ assimilation capacity. CO_2_ assimilation acts as a major sink for the reducing equivalents (ATP and NADPH) generated by the primary photochemical reactions.

Relative reflectance is also an index reflecting different stresses. Several indices have been used for estimating chlorophyll or nitrogen content (Yendrek et al., 2017). For plants exposed to nitrogen deficiency, the synthesis of chlorophyll is hindered, resulting in the change of leaf spectral distribution (Kuckenberg et al., 2009; Bürling et al., 2011). We use relative reflectance 550/510 to represent the change of leaf chroma. As nitrogen content decreases, the inhibition of chlorophyll synthesis will cause decline of relative reflectance 550/510 (**Figures 5, 6A**). However, during the early stage of drought stress, the inhibition of chlorophyll synthesis will not be obvious and the relative reflectance 550/510 will not decline (**Figure 4A**). The different changing pattern of 550/510 values under nitrogen deficiency as compared to other stresses can be used to identify the occurrence of nitrogen deficiency.

Pathogens acquire nutrient through interfering the plant metabolism, resulting in similar changes in chlorophyll fluorescence and multispectral signals as the nitrogen deficiency (Rolfe and Scholes, 2010; Ivanov and Bernards, 2016). However, the development rate of pathogen infection is fast, and plant leaves will curl downwards. To distinguish these two stresses, the analysis based on images should not be ignored (Garciaruiz et al., 2013; Wetterich et al., 2016).

### Attention About the Monitoring Method

Statistics analysis about the indicator images can be carried out for drought and nitrogen deficiency stress. However, plant disease has the characteristic of sudden incidence and heterogeneity of infection area. Statistics may cover the disease/healthy area. Thus, analysis based on images should not be ignored. On the other hand, overall growth condition of the crop is more important in practical agricultural production. If the infection area is small and local, that means the disease doesn’t affect the crop broadly yet. Before the disease is spreading out, effective management should be taken.

There are also some other possibilities that affect the plant photosynthesis. In the process of agriculture production, apart from the Φ_PSII_ decrease caused by drought stress, another common stress is temperature fluctuation. This will cause interference to the judgment of plant physiology. However, in practice the temperature factor can be excluded by temperature recording.

## Conclusion

We built a high throughput crop physiology monitoring method based on chlorophyll fluorescence and multispectral imaging in this study. This system can distinguish typical crop stresses such as drought, nutrition deficiency and plant disease in a simple way. Meanwhile we have studied the correlation between the physiological indicators and the stresses, which makes the degree of stress can be estimated. This method can provide basis for crop management, and furthermore provide possibility of automatic machine diagnosis.

## Author Contributions

HL, XX, LD, JY, and XL contrived the study. HW, SX, and LX built the monitoring system. HW, XQ, LZ, and SX performed the experiments. HW and SX analyzed the data. HW, LZ, and XX interpreted the results. HW and LZ wrote the manuscript. HL and XX made revisions.

## Conflict of Interest Statement

The authors declare that the research was conducted in the absence of any commercial or financial relationships that could be construed as a potential conflict of interest.

## Footnotes

1 http://www.walz.com/

2 http://www.psi.cz/

3 http://www.hansatech-instruments.com/

## Supplementary Material

FIGURE S1 The physiology monitoring system with chlorophyll fluorescence module and multispectral module **(A,B)** and the scene when the system is working **(C)**.

FIGURE S2 Φ_PSII_, *F*_v_/*F*_m_, and 550/510 pseudo color images and photos of tomatoes under drought stress.

FIGURE S3 Φ_PSII_, *F*_v_/*F*_m_, and 550/510 pseudo color images and photos of tomatoes in drought stress control group.

FIGURE S4 Φ_PSII_, *F*_v_/*F*_m_, and 550/510 pseudo color images and photos of tomatoes under nitrogen deficiency stress.

FIGURE S5 Φ_PSII_, *F*_v_/*F*_m_, and 550/510 pseudo color images and photos of tomatoes in nitrogen deficiency stress control group.

FIGURE S6 Φ_PSII_, *F*_v_/*F*_m_, and 550/510 pseudo color images and photos of tomatoes infected with *Botrytis cinerea*.
