# Supplementary figures and images for: A Method of High Throughput Monitoring Crop Physiology Using Chlorophyll Fluorescence and Multispectral Imaging
Source: Front Plant Sci. 2018 Mar 28;9:407. doi: 10.3389/fpls.2018.00407 (PMC5883069; doi:10.3389/fpls.2018.00407)

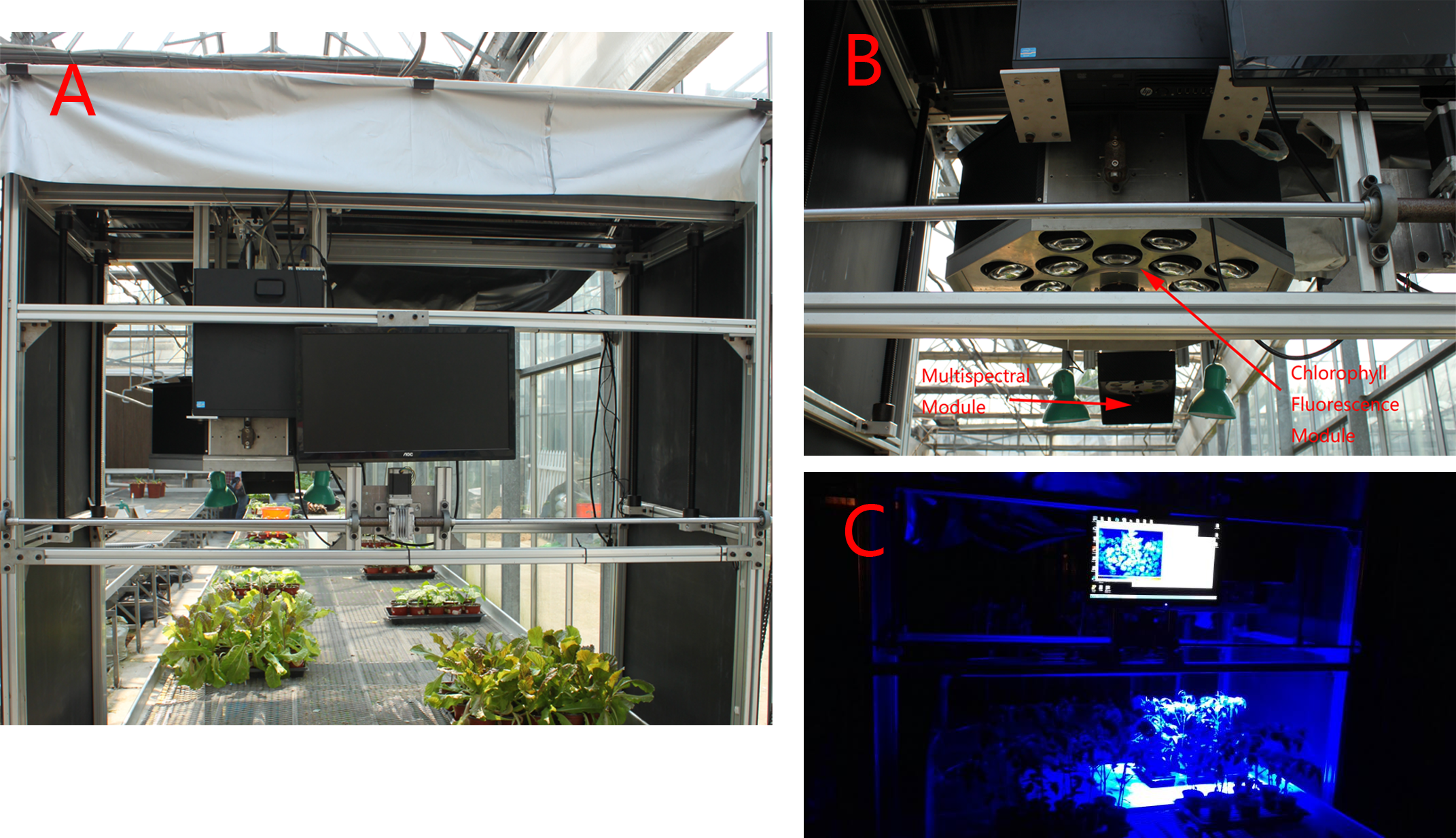

Supplement: FIGURE S1 — The physiology monitoring system with chlorophyll fluorescence module and multispectral module (A,B) and the scene when the system is working (C). [file Image_1.TIF]

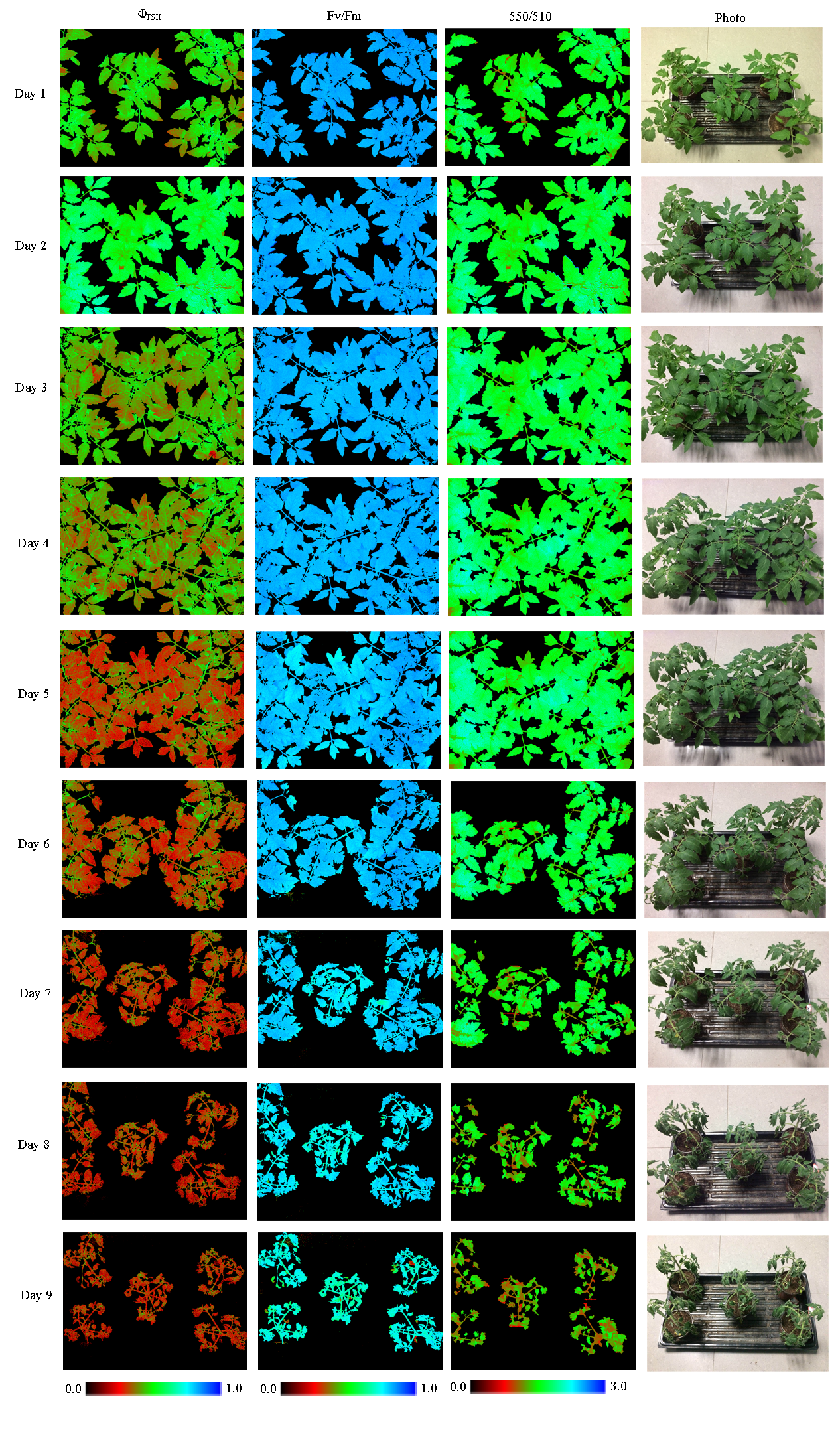

Supplement: FIGURE S2 — ΦPSII, Fv/Fm, and 550/510 pseudo color images and photos of tomatoes under drought stress. [file Image_2.TIF]

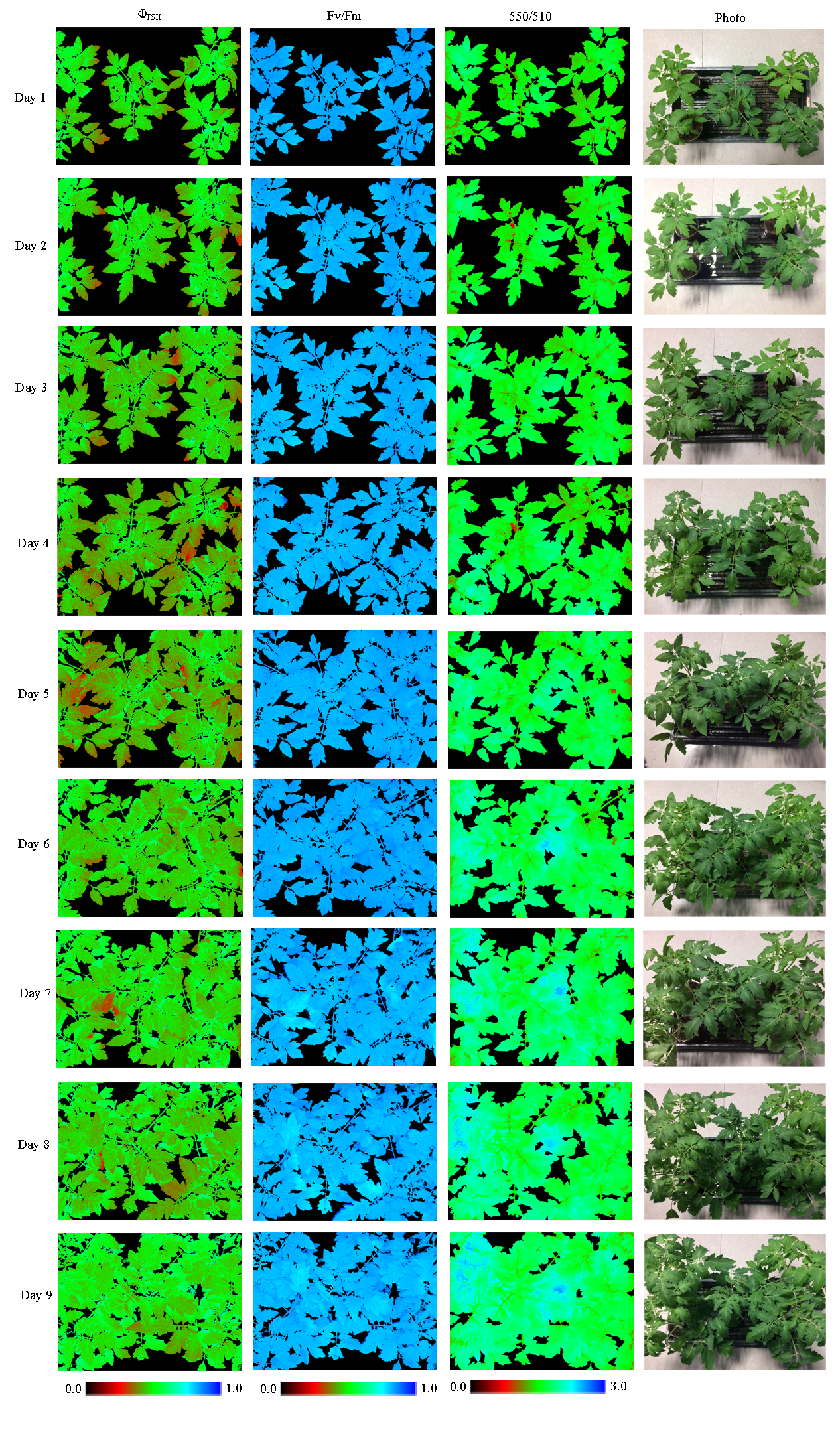

Supplement: FIGURE S3 — ΦPSII, Fv/Fm, and 550/510 pseudo color images and photos of tomatoes in drought stress control group. [file Image_3.TIF]

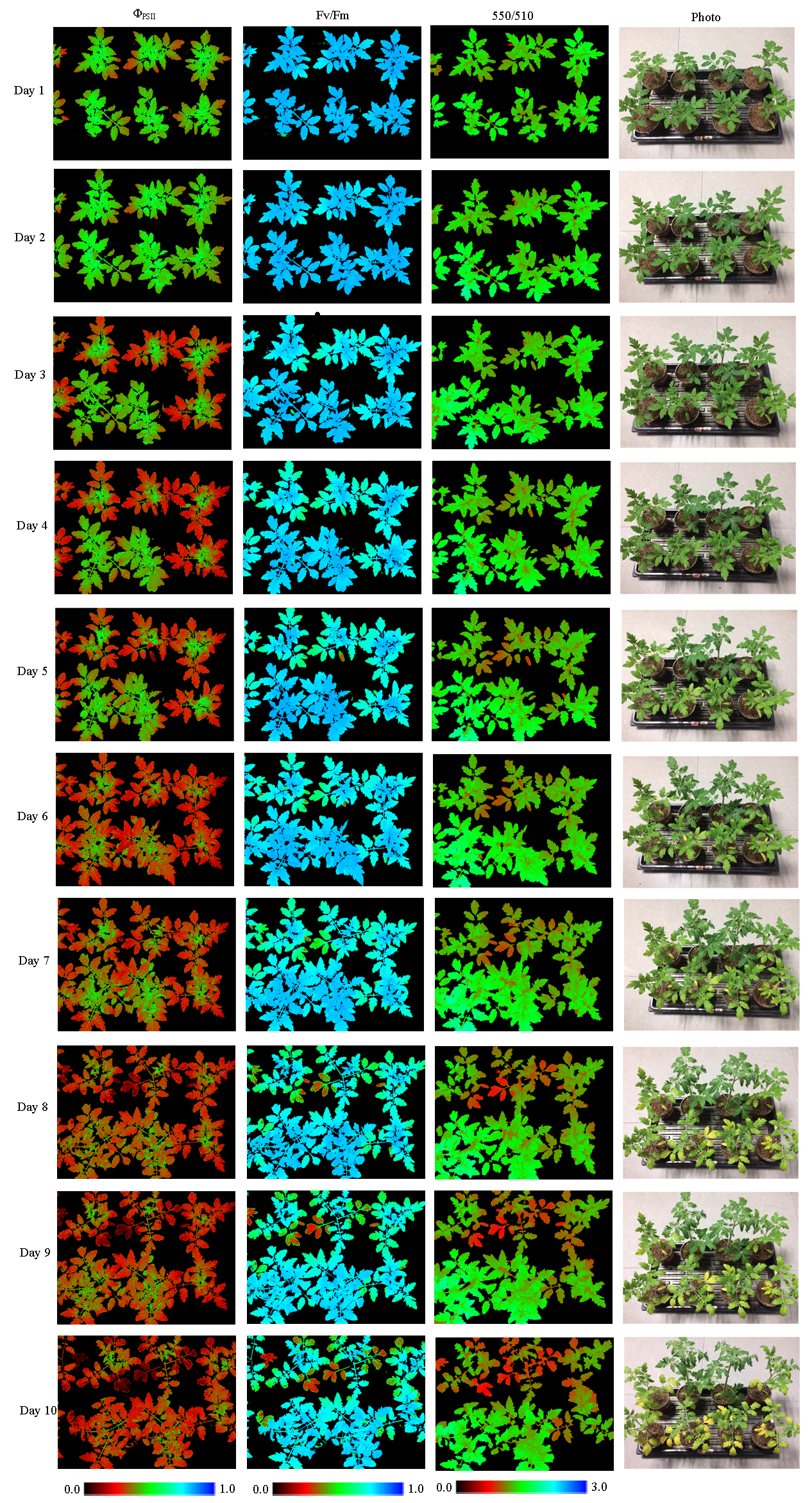

Supplement: FIGURE S4 — ΦPSII, Fv/Fm, and 550/510 pseudo color images and photos of tomatoes under nitrogen deficiency stress. [file Image_4.TIF]

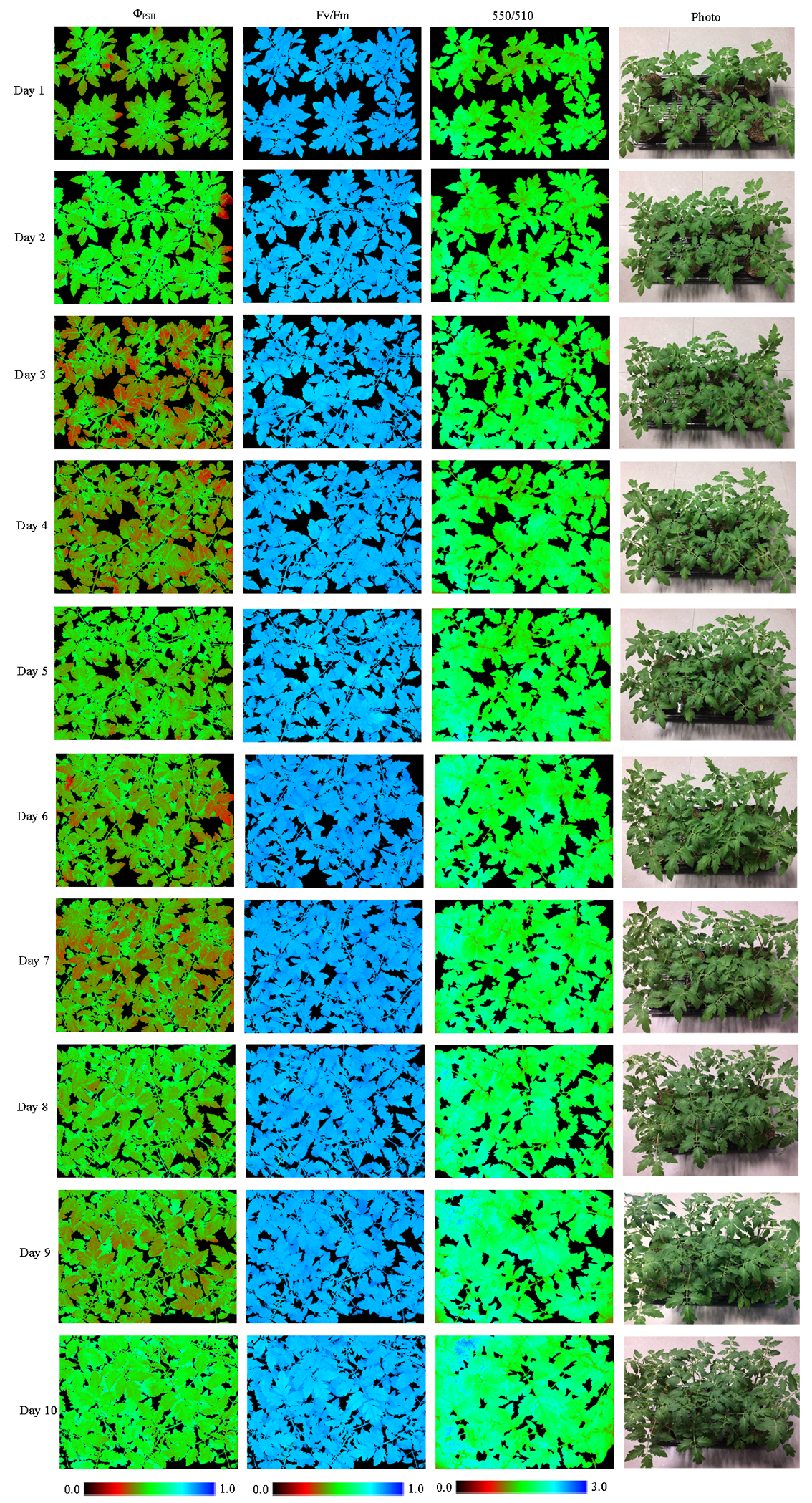

Supplement: FIGURE S5 — ΦPSII, Fv/Fm, and 550/510 pseudo color images and photos of tomatoes in nitrogen deficiency stress control group. [file Image_5.TIF]

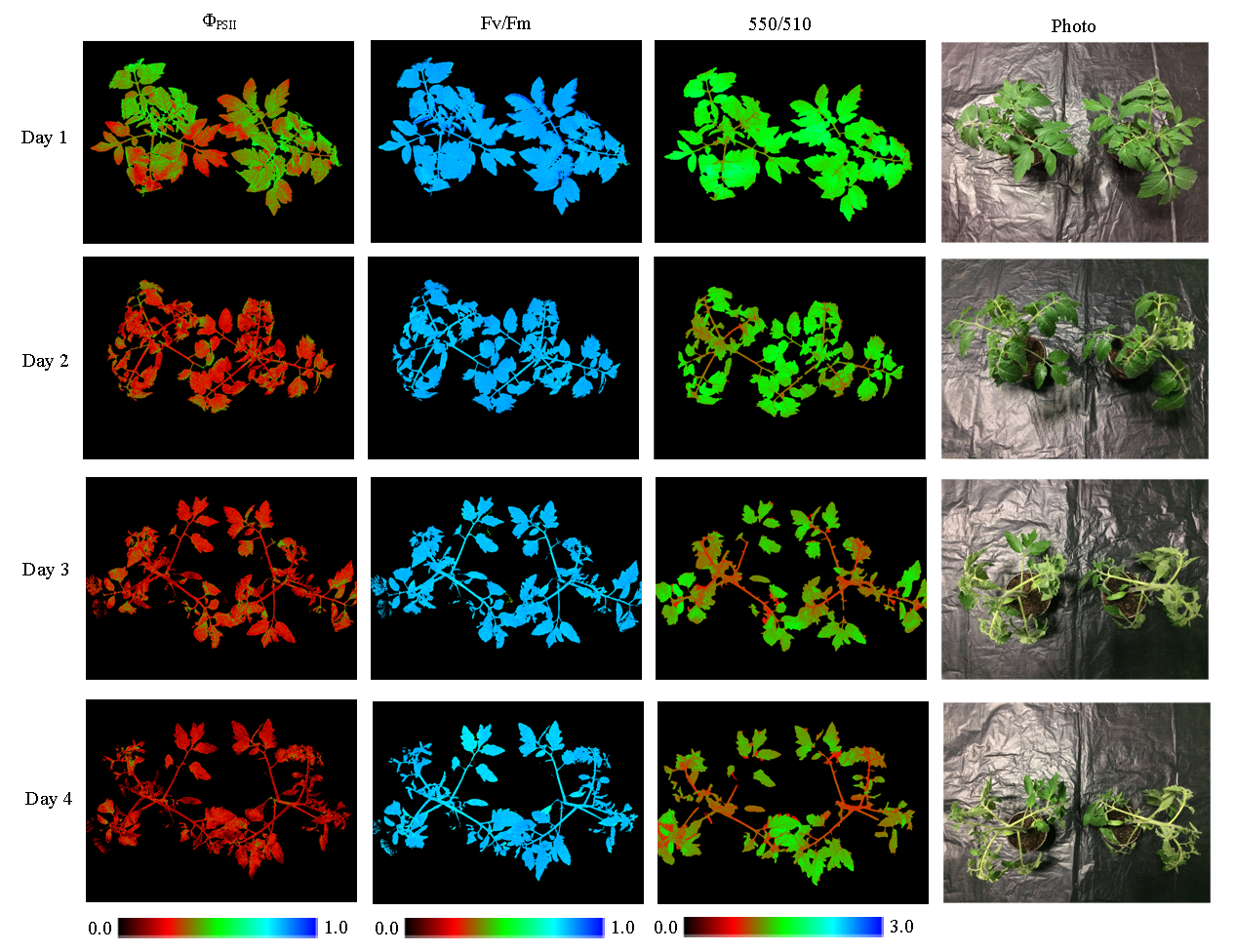

Supplement: FIGURE S6 — ΦPSII, Fv/Fm, and 550/510 pseudo color images and photos of tomatoes infected with Botrytis cinerea. [file Image_6.TIF]
